# Reinforcement Learning-Based BEMS Architecture for Energy Usage Optimization

**DOI:** 10.3390/s20174918

**Published:** 2020-08-31

**Authors:** Sanguk Park, Sangmin Park, Myeong-in Choi, Sanghoon Lee, Tacklim Lee, Seunghwan Kim, Keonhee Cho, Sehyun Park

**Affiliations:** School of Electrical and Electronics Engineering, Chung-Ang University, Seoul 06974, Korea; pppssuu@cau.ac.kr (S.P.); motlover@cau.ac.kr (S.P.); auddlscjswo@cau.ac.kr (M.-i.C.); leessan0@cau.ac.kr (S.L.); tacklim34@cau.ac.kr (T.L.); tkftn456@cau.ac.kr (S.K.); thckwall@cau.ac.kr (K.C.)

**Keywords:** reinforcement learning (RL), artificial intelligence (AI), building energy management system (BEMS), energy optimization, internet of things (IoT)

## Abstract

Currently, many intelligent building energy management systems (BEMSs) are emerging for saving energy in new and existing buildings and realizing a sustainable society worldwide. However, installing an intelligent BEMS in existing buildings does not realize an innovative and advanced society because it only involves simple equipment replacement (i.e., replacement of old equipment or LED (Light Emitting Diode) lamps) and energy savings based on a stand-alone system. Therefore, artificial intelligence (AI) is applied to a BEMS to implement intelligent energy optimization based on the latest ICT (Information and Communications Technologies) technology. AI can analyze energy usage data, predict future energy requirements, and establish an appropriate energy saving policy. In this paper, we present a dynamic heating, ventilation, and air conditioning (HVAC) scheduling method that collects, analyzes, and infers energy usage data to intelligently save energy in buildings based on reinforcement learning (RL). In this regard, a hotel is used as the testbed in this study. The proposed method collects, analyzes, and infers IoT data from a building to provide an energy saving policy to realize a futuristic HVAC (heating system) system based on RL. Through this process, a purpose-oriented energy saving methodology to achieve energy saving goals is proposed.

## 1. Introduction

To help realize a worldwide sustainable society by saving energy in new and existing buildings, many intelligent building energy management systems (BEMSs) are emerging. BEMS refers to a system that integrates the internet of things (IoT) technology into a building to manage multiple building facilities [1,2,3]. A BEMS aims to create a pleasant environment by managing the various facilities used in buildings to save energy, reduce labor costs, and extend the lifetimes of the buildings [4]. BEMS in buildings that had smart IoT technology incorporated during construction are called new BEMSs, and BEMS added to newly remodeled existing buildings are called existing BEMSs. Currently, new BEMSs into which smart IoT technology is integrated from the planning stage of building construction are emerging. However, IoT technology is also being integrated into existing BEMSs to create new energy-saving smart BEMSs. Research is underway to improve energy efficiency by eliminating wasteful energy elements throughout existing buildings [5].

To implement a BEMS in a new building, an IoT-based intelligent system can be installed from the pre-planning stage at low cost. It is not problematic to select and apply high-efficiency building equipment in the early stages of construction and to construct the latest IoT-based HVAC and integrated management system. However, implementing a BEMS in an existing building involves higher costs, such as replacing expensive facilities and hiring additional building managers to apply the smart IoT system. The wastage of energy can be reduced by replacing obsolete and expensive facilities with BEMS retrofits in existing buildings; however, the most reasonable option is to install and apply light and low-cost IoT [6,7,8] devices inside the building to monitor environmental and energy usage information. It is reasonable to increase the energy operation efficiency in this way because of the high costs (price, size, labor cost) involved in building a BEMS in an existing building.

The first important concept here is that of a light and cost-effective IoT device [9]. To build a BEMS, IoT devices are attached to the building’s surface (highest layer) to perform important tasks. In other words, IoT devices are installed on the building in high proximity to the user and the environment, and they do not require the replacement of heavy and expensive equipment to implement a BEMS in an existing building. In addition, IoT sensors and control interfaces are installed on the surface of the facility to collect user and environmental information and to intelligently control the facility based on this information. The second important aspect of the IoT is the use of large amounts of data. By analyzing user and environmental information from large amounts of data collected from IoT devices over a period of time, the most efficient guidelines for saving energy can be applied to the HVAC system. The HVAC system references guidelines based on the analyzed data to provide an optimal HVAC environment for the user while reducing energy. Currently, artificial intelligence (AI) is the most extensively used state-of-the-art technology for data analysis [10,11].

### 1.1. AI-Based Energy Management

The main data analysis methods proposed in this paper are machine learning (ML) and reinforcement learning (RL). ML is a type of AI that is part of the field of data science. Data science is a multi-disciplinary field in which knowledge and insights are extracted from structured and unstructured data [12,13,14,15,16]. Data science unifies statistics, data analysis, ML, and related methods to understand and analyze actual phenomena using data [17]. AI is a typical area of data science [18]. ML and deep learning are key factors in AI, and this study uses RL technology, which is part of ML. The basic principle of RL is learning, which is the ability to determine rules from given data and algorithm-based intelligence [19]. A BEMS should recognize correlations in the data (User-HVAC) that are generated in the building through learning and determine where to apply this knowledge to obtain rewards. Although a number of studies have been conducted on implementing an ML-based BEMS, it has only been used as a means of predicting the future based on a simple data analysis and applying the results. However, to fundamentally apply RL to building energy management, it is necessary to identify the relationships with regard to the HVAC system inside the building and apply that information to the actual situation. This is a very important point, so we present the overall structure for applying RL to the building. The related explanations are presented in Section 3, Section 4 and Section 5.

### 1.2. Intelligent Energy Data Analysis

In this study, ML-based reasoning models were applied to the analysis of time series data collected from sensors. By analyzing past temperature sensor data, we inferred the temperature data figures for the next two days or more to establish and provide a guideline policy on how to drive the operation of the HVAC system [20]. Time series data refers to data that are represented as a function of time. Examples of time series data include yearly sales values for the past five years and typhoon positions every three hours, and these data are mainly used for prediction and inference for a certain period of time in the future [4,21]. This study analyzes the time series data from real-time user and building information collected by IoT sensors installed in the building. The data to be analyzed are mainly temperature measurements, corresponding to the time series data. By analyzing the trends in temperature changes in the user’s room with respect to time, it is possible to control the future operations of the HVAC system. By inferring the trend in future temperature changes through the analysis of time series data, the operation policy of the HVAC system can be adjusted accordingly.

### 1.3. Purpose-Oriented Energy Saving and Optimization

This paper proposes a “purpose-oriented energy saving plan” to save energy on building HVAC systems. Purpose-oriented energy savings refers to pre-setting the amount of energy savings that have been achieved and then establishing energy saving policies. For example, assuming that the maximum energy savings available to date for the current BEMS are 11%, the proposed system will conclude that 25% of energy savings have already been achieved. For example, if the HVAC system that consumes the most energy in the existing system brought an energy savings of 11% through the pruning of wasted elements, the proposed plan is to drastically reduce the operating rate of the HVAC system by 25%. In conclusion, it is a method of achieving an energy savings of 25% of the total energy use of the HVAC system. The salient point is that the energy use of the HVAC system should be reduced by 25%, and the corresponding reduction in heating should be made according to the user’s needs and analyzed data. This method causes concern that the HVAC system’s QoS (quality of service) will be reduced for the users. However, it will be controlled by converging the temperature setting history and various complaints of the building users. That is, this method provides the best alternative for satisfying the two conditions (achieving energy savings at the same time as satisfying the user’s QoS) by identifying the time zones that do not affect the users of the HVAC system and using that information to reduce the energy used.

### 1.4. The Purpose of This Study

This study is based on the four methods described above. To implement an IoT-based BEMS more efficiently and intelligently target IoT-based energy-saving systems that are difficult to apply to a current base building, a light and inexpensive IoT sensor is attached to the building surface to collect user and environmental information. The collected data is then analyzed to provide intelligent services in the future, whereby the user benefits from optimal temperature maintenance in the building, to achieve optimal HVAC (heating) system energy savings. That is, data collected from the IoT sensor is analyzed and, based on ML, used to provide the optimal temperature at the desired time for the user. Thus, the energy use of the building is based on RL to reflect the needs of these users. In other words, in this study, the user is provided with optimal conditions at the same time the energy use of the HVAC system is reduced to optimize the building with four energy models. The main scope and procedure of the study can be summed up as follows:

- **Main scope**
  1. Smart IoT-based cost-effective BEMS for existing buildings
  2. RL-based energy management model
  3. ML-based data analysis for time-series data reasoning
  4. Purpose-oriented energy saving and optimization methodology based on RL
- **Total procedure**
  1. **Step 1:** Collecting data by installing a smart IoT device inside the building (Section 4).
  2. **Step 2:** Establishing the relationship between the user and the building based on RL (Section 3).
  3. **Step 3:** Inferring the collected data based on ML (Section 5).
  4. **Step 4:** RL-based building energy optimization by establishing an HVAC schedule to be applied in the future based on the inferred data (Section 6).

First, in Section 2, related works, the key scenarios associated with this paper are analyzed and the shortcomings of existing system and benefits of proposed system are explained. In Section 3, system architecture, and Section 4, system configuration, the overall system architecture is presented, including the flow chart and scenarios of the system. In the simulation results, the simulation results of the actual analysis (Section 5) and energy optimization are provided (Section 6). Finally, in the last section, the business model, future prospect, and final conclusions are presented.

## 2. Related Works

### 2.1. ANN (Artificial Neural Network)-Based BEMS Modeling

Zhao, et al. [22] described the recent studies, including engineering, statistical, and artificial intelligence based studies, related to modeling and forecasting building energy consumption. In the aforementioned paper, it was suggested that the most widely used artificial intelligence methods are artificial neural networks (ANNs) and support vector machines (SVMs). Kalogirou, et al. [23] suggested various applications of neural networks in relation to energy systems. This demonstrates the function of artificial neural networks as a tool for energy prediction and modeling in energy-related fields. Chae, et al. [24] proposed a short-term building energy usage prediction model based on an artificial neural network (ANN) using a Bayesian normalization algorithm and elucidated the effects of network design parameters such as time delay, number of hidden neurons, and training data on the performance of the model. Kalogirou, et al. proposed this paper [25] was to create a simulation program that uses an ANN to model the thermal behavior of buildings by employing a multilayer iterative architecture using a standard back propagation learning algorithm.

### 2.2. Reinforcement Learning (RL)-Based BEMS Modeling

Liu, et al. [26,27] summarize the RL techniques used to control building heat storage. Yu, et al. [28] proposed a model-less method based on the RL scale to coordinate the supervisory controllers of energy-saving building systems online. Therein, it was proposed that the supervisor learns the optimal value of one parameter and selects the most suitable rule set based on the fuzzy rule set generated by offline optimization, thereby reducing the learning time and computational requirements. Kantamneni, et al. [29] investigated the application of a multi-agent system (MAS) in the control and operation of microgrids. An MAS consists of a single agent or multiple intelligent agents that interact to solve problems beyond the capabilities of the system. This paper discussed MAS concepts, architecture, platform and process development, sample application provision, and limitations. This architecture is similar to RL that is based on using simple agents in the environment. Dimeas, et al. [30] proposed a general framework for microgrid control based on a multi-agent system technology. The proposed architecture can integrate several functions to match the complexity and size of the microgrid. To achieve this, the idea of hierarchical learning is used, and various controls and actions of agents are grouped according to their impact on the environment. It also focused on how agents work together to achieve their goals. At the heart of the collaboration is a multi-agent RL algorithm that allows the system to operate autonomously in island mode. Vázquez-Canteli, et al. [31] presented an integrated simulation environment combining CitySim, a high-speed building energy simulator, and TensorFlow, a platform for the efficient implementation of advanced ML algorithms, in smart city environments. Brandi, et al. [32] proved that deep reinforcement learning (DRL) can contribute to significant energy savings in building environmental heating systems. A DRL-based heating system in an office environment was proposed; energy savings of 5% and 12% were achieved by the proposed energy saving scenarios.

### 2.3. Intelligent BEMS for Energy Optimization

Wang, et al. [33] proposed a pre-multipurpose optimization model for BEMS by integrating solar power generation with other power generation methods as a source of economic and resident comfort. User comfort includes three aspects with regard to the indoor environment: visual comfort, thermal comfort, and indoor air quality comfort. By considering the controllable loads that can participate in the demand and response (DR) program, a balance between various energy styles, electrical, thermal, and cooling loads is ensured during the optimized operation. MATLAB’s YALMIP toolbox was applied to solve the optimization problem, and a case study was conducted to verify the effectiveness and adaptability of the proposed model. Wang, et al. [34] proposed an occupancy-related energy-cyber-physics system incorporating Wi-Fi probe-based occupancy detection. The proposed framework extracts three types of occupancy information using an ensemble classification algorithm. It enables automatic occupant detection and interpretation by creating a data interface to connect energy management and cyber physics systems and assembling several weak classifiers for Wi-Fi signals. To investigate the performance of the proposed occupied energy-cyber-physical system, verification experiments were conducted in large office spaces, and it was found that the system saved energy by approximately 26.4%. Degha, et al. [35] proposed an intelligent context-aware building energy management system (ICA-BEMS). The ICA-BEMS uses a hybrid energy saving technology based on smart context-awareness management (Smart-CAM). Smart-CAM uses smart building ontology to gather smart building knowledge and provide contextual information using new context awareness mechanisms. Park, et al. [36] presented that a human comfort-based control approach for intelligent DR-based home energy management. The heating and lighting system was controlled by the elements of thermal comfort and visual comfort. As a result, this paper showed that the proposed approach can effectively reduce energy consumption and improve user comfort. Machine learning is a technology that is effectively applied to intelligent energy saving in BEMS. Dey, et al. [37,38,39] shows optimized energy saving and user convenience through machine learning based fault detection and diagnosis. Jafarinejad, et al. [40] proposed a bi-level energy-efficient occupancy profile optimization method integrated with a demand-driven control strategy to optimize the energy consumption within in a university departmental building.

### 2.4. Novel Energy Saving Routing Algorithm with Q-Learning Algorithm

Zhang, et al. [41] researched MANET (mobile ad hoc network), VANET (vehicular ad-hoc network) by the heuristic Q-Learning algorithm can dynamically adjust the routing path through interaction with the surrounding environment. Zhang, et al. [42] proposed an energy-balanced routing method based on forward-aware factor. The next-hop node is selected according to the awareness of link weight and forward energy density, and a spontaneous reconstruction mechanism for local topology is designed additionally.

Thus far, many papers related to AI-based intelligent modeling and intelligent BEMS energy optimization have been published. Although there are numerous studies related to building energy optimization, they have several limitations. Table 1 shows the limitations of existing systems and the methods used by the system proposed herein to solve them.

### 2.5. Problems of Existing System

1. Problem 1: Simple data prediction system using AI: Simple data analysis is only suitable for weather predictions, such as temperature and fine dust predictions.
2. Problem 2: Energy savings via simple facility replacements for existing buildings: It is necessary to replace existing low-efficiency, old facilities to build BEMSs in existing buildings. However, these methods are expected to be expensive and difficult to install and require additional installation costs.
3. Problem 3: Passive energy saving measures: There is a lack of precise goal setting for energy saving.

This proposed system has the following advantages that effectively address the aforementioned problems:

### 2.6. Merits of the Proposed System

1. Solution 1: Reinforcement learning (RL)-based building energy optimization: This represents a more advanced AI-based method that functions via interactive exchanges between the RL-based user and HVAC.
2. Solution 2: IoT-based lightweight and cost-effective system for existing buildings: Energy management is performed by installing a lightweight and cost-effective IoT system rather than replacing expensive equipment to build BEMSs in existing buildings.
3. Solution 3: Purpose-oriented energy saving schedules: Energy-saving plans that are active, rather than passive, are proposed. Existing energy-saving methods are insufficient for achieving building energy savings up to 25%. Purpose-oriented energy saving plans means assuming that a 25% energy savings has already been achieved and then gradually complementing the user’s complaints by controlling the HVAC.

## 3. System Architecture

### 3.1. RL-Based System Architecture

**Figure 1:**
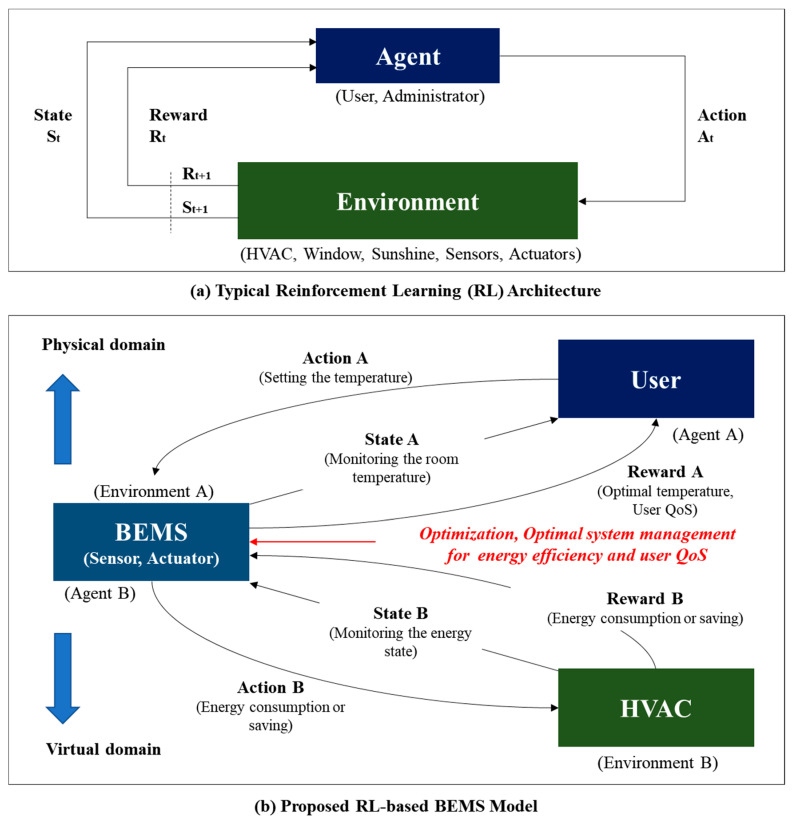
Reinforcement learning (RL)-based BEMS architecture.

Figure 1a shows the basic structure of the RL architecture. It consists of the agent and environment, as shown in the Figure 1. The main body is called the agent. The factors it interacts with, comprising everything other than the agent, is called the environment. The agent selects actions, and the environment responds to these actions, continually presenting new situations to the agent. The environment also provides rewards, which are special numerical values that the agent seeks to maximize over time through its choice of actions, to the agent [43,44].

In this study, we installed a smart IoT system for a real hotel building based on the RL architecture and implemented a RL-based BEMS, as shown in Figure 1b. Figure 1b shows the RL architecture applied to the BEMS. As shown in the figure, the system is structured to receive rewards via user-conditioning system interactions. The following are possible relationships that can form between the agents and the reward in the hotel. The term agent includes users, administrators, and cleaners. The environment includes the HVAC system, windows, sunlight, and sensors. The rewards are user QoS and energy savings. The final goal is optimal system management with regard to energy efficiency and user QoS. In this study, there are two aspects, as shown in Figure 1b. In other words, when the agent is the user and the environment is the HVAC system, the reward will be the user QoS. On the other hand, when the agent is the BEMS system and the environment is the HVAC, the reward is energy consumption and saving energy efficiency. The following figure presents a more detailed explanation.

As shown in Figure 1b, Box 1 and Table 2, the overall structure is divided into two parts: the user-BEMS side and the BEMS-HVAC system side. Both aspects are considered in this paper.

Box 1 **Main procedure of RL-based BEMS model.**

**Main procedure**

**Step 1:** Establish RL-based BEMS architecture

**Step 2:** Connection of RL-based main factor and BEMS factor

**Step 3:** Analysis of relationship of user-BEMS side and BEMS-HVAC system side

- User-BEMS side
  - Action A (User request)
  - Reward A (User Satisfaction or user dissatisfaction)
- BEMS-HVAC system side
  - Action B (Energy consumption)
  - Reward B (Energy saving)

**Step 4:** Optimization

#### 3.1.1. User-BEMS Side

**Figure 2:**
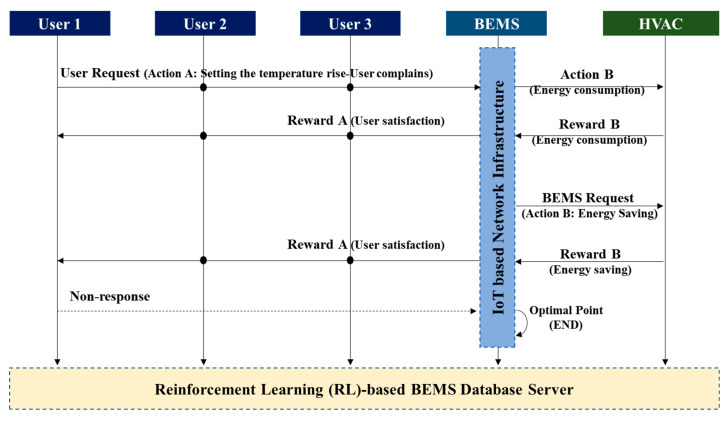
Agent–environment interaction in an RL-based BEMS.

First, the user becomes an “agent” and affects the BEMS, which is the “environment.” The agent has a target for the BEMS: to increase the user’s QoS. That is, the user, who is the agent, commands the BEMS to raise or lower the temperature to increase his/her satisfaction. This is called the “User Request” in Figure 2. If the user asks the BEMS to raise the temperature, the BEMS will raise the temperature to give the user a high QoS as a reward. This is the structure of a general heating system.

#### 3.1.2. BEMS-HVAC System Side

However, in this study, we approached it from a different perspective and considered the second side. This is the situation in which the BEMS acts as the agent. If the BEMS is the agent, the HVAC system is the environment. The BEMS has one purpose: to save energy. For the purpose of saving energy, the BEMS requests energy savings from the user, the environment. This is called the “BEMS Request” in Figure 2. The BEMS asks the HVAC system for energy savings. However, rather than making a request, the BEMS saves energy by arbitrarily lowering the temperature at a specific time period based on the user’s past temperature-setting history. This time is selected based on when the user has decreased the temperature or ventilated in the past. That is, a user in the past turned off the HVAC system or selected a point at which the temperature dropped sharply through ventilation, thereby ceasing the operation of the HVAC system at that time. As this action is inferred and performed based on past data rather than being requested by the user in real-time, the user’s QoS will be degraded. However, the HVAC system does not consider this and arbitrarily reduces its use. Thus, the BEMS achieves the goal of energy saving.

#### 3.1.3. Optimization

The RL-based BEMS database server stores the data history between all users and the HVAC system and establishes an HVAC policy through data analysis. The problem is resolving the QoS of users when the HVAC system proceeds in this manner. The solution is to consider the user’s request or complaint. If the user feels cold owing to a temperature drop, the user must raise the temperature or complain to the manager through the interface of the HVAC system. When the complaint occurs, the temperature is raised during the next scheduling. If a complaint does not occur, the state is determined by the user to be at an optimum temperature, and the temperature is not raised. Thus, the “optimization” in Figure 1b determines the optimal point by converging the user and BEMS requests. In this paper, a method is proposed to find the most optimal compromise and to save energy in the HVAC system while maintaining the user’s satisfaction.

## 4. System Configuration Test-Bed

### 4.1. System Installation

A smart IoT system was installed in hotels located in South Korea to implement the RL-based purpose-oriented BEMS. The first was a pre-analysis of the “S” hotel, and an IoT system was designed and built for that location. Table 3 shows the main analysis information of the hotel as well as a blueprint for a smart IoT system installed on each floor, along with an analysis of the overall facilities of the “S” hotel. Table 4 is a description of the IoT system installed therein.

The hotel consists of a total of 26 floors, as shown in Table 3. The total number of rooms is 383, and heat is supplied to each room through two central boilers. The boilers can operate for 24 h a day, and their uptime is adjusted according to the weather. Each room has a pan-coil for opening and closing the duct slot on the ceiling of the room, providing centralized heat energy to each room. That is, the user controls the temperature of the room via the interface in the room. Inside the room, a smart IoT sensor and a gateway collect the environmental information from the room (temperature/humidity, power, motion, etc.), as shown in Table 4. A detailed description of this is presented in Section 4.2.

### 4.2. System Configuration

**Figure 3:**
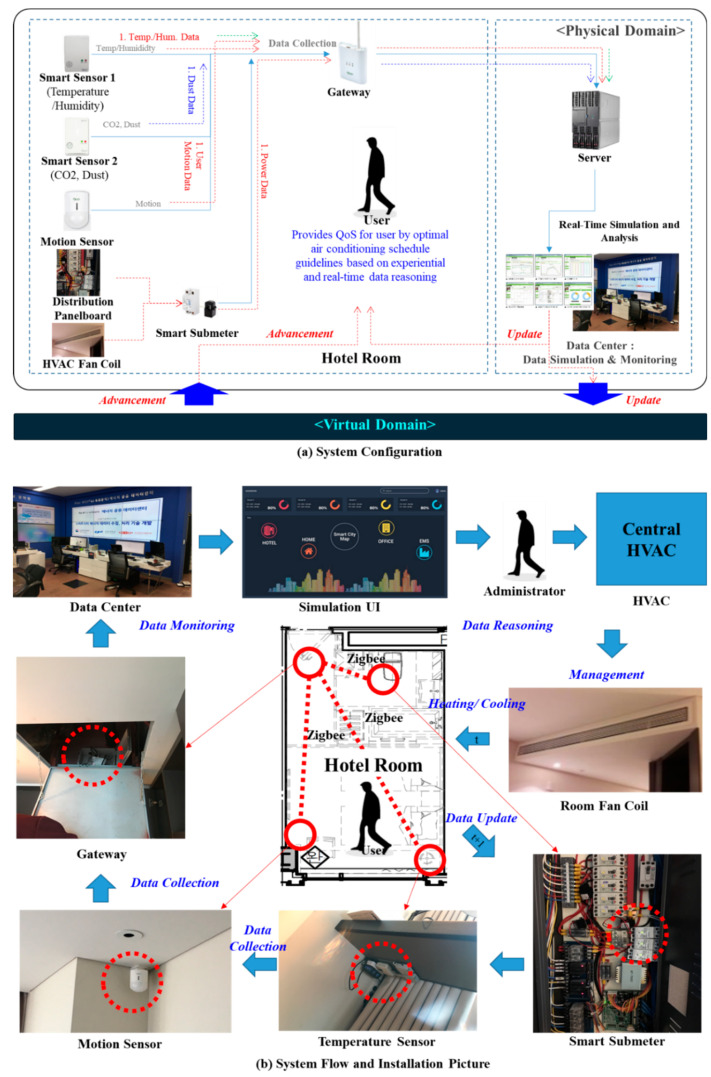
System Configuration.

The Figure 3 represents the structure of the IoT system actually installed in the hotel environment. All IoT sensors used communicate based on Zigbee, which is IEEE 802.15.4 standard-based wireless network technology; real data transmission rate was measured and confirmed to be over 2.5 kbps (ACK transmitted ≈ ACK received). Data sampling rate is ‘1 sampling/min’. This cycle is changeable; we collected data by setting it to the most reasonable ‘1 sampling/min’ considering the battery consumption period. Figure 3a represents the installed IoT system structure; the entire system is composed of a sensor (temperature/humidity, CO_2_, fine dust sensor), actuator (HVAC system), and data center. The data center collects the temperature/humidity, CO_2_, fine dust, motion detection data, and power sensor data from the power portion of the fan coil. The collected data is transmitted through the gateway via a Zigbee-based wireless communication protocol and is transmitted to the central server of the data center. The temperature and humidity data are transmitted from each room by the temperature/humidity sensor and the air quality data in the room is transmitted by the CO_2_ and fine dust sensors. The movement of the users in the room is detected by the motion detection sensor, and the power information is collected and transmitted by the smart submeter installed in the power portion of the fan coil of the in-room duct responsible for the HVAC heating and cooling. The power information from the fan coil can be measured whether the HVAC system in the room is activated. Figure 3b shows pictures of the actual IoT devices installed inside the hotel.

### 4.3. System Flow and Scenarios

**Figure 4:**
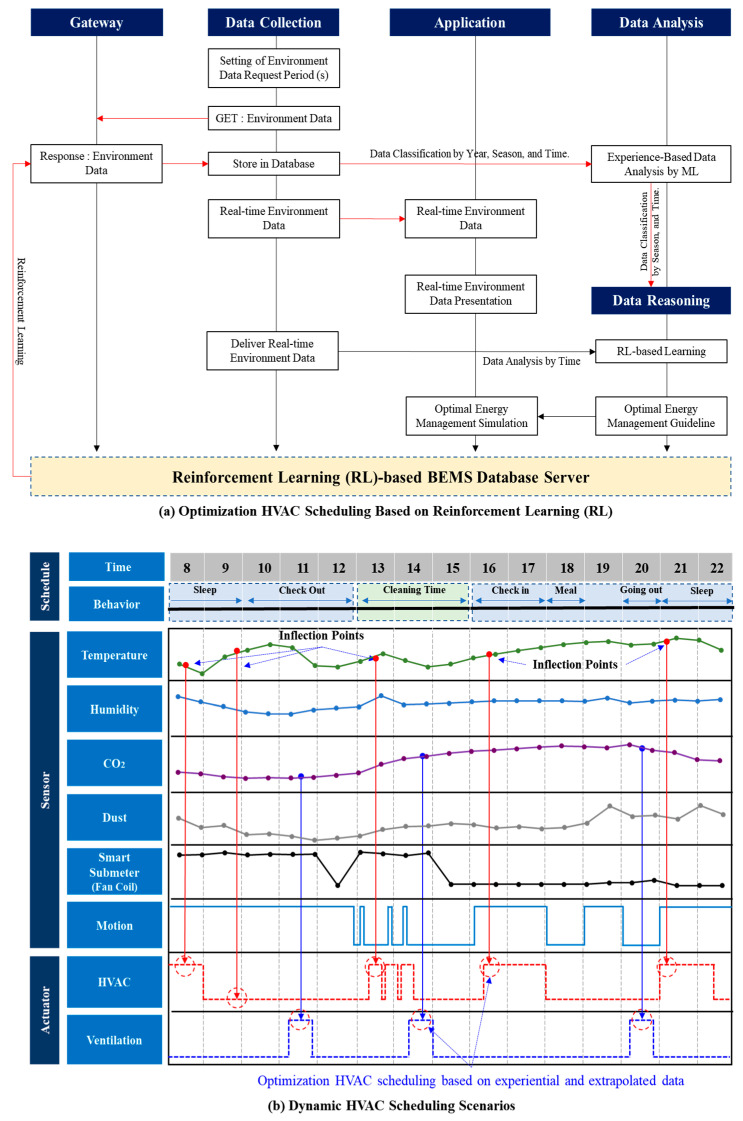
System Flow and Scenarios.

Figure 4a shows the flow chart of RL-based HVAC optimization. When the temperature/humidity, air quality, user movement, and power information from the fan coil are collected from the sensor, they are transmitted to the data center via the gateway. The data center can monitor these data in real time and collect and classify data hourly, seasonally, and annually to intelligently infer future progress. This will create an energy-saving policy for future energy savings. Based on this, the administrator carries out the energy saving policy by setting the HVAC schedule when the user requests a temperature increase. As shown in Figure 4b, the HVAC schedule is performed during heating when the temperature rise history is high, when the fan coil operation history is high, and when the user movement is high. A detailed explanation is given in Section 5.

## 5. Data Analysis

### 5.1. Temperature Data Analysis

This study implements optimal control scenarios for energy savings in heating systems by analyzing the temperature rise history when heating hotels in the scenario. Box 2 shows a data analysis procedure.

Box 2 **Data analysis procedure.**

**Data analysis procedure**

**Figure 5:**
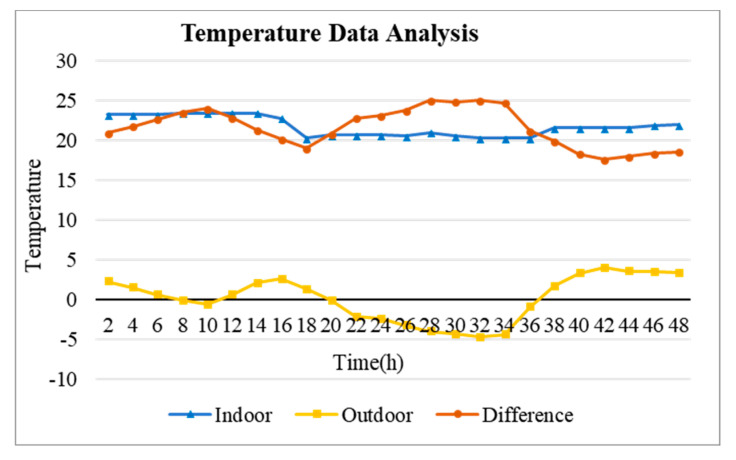
Temperature Data Analysis.

**Step 1:** Analyzing the temperature data of the hotel rooms (Figure 5)

**Figure 6:**
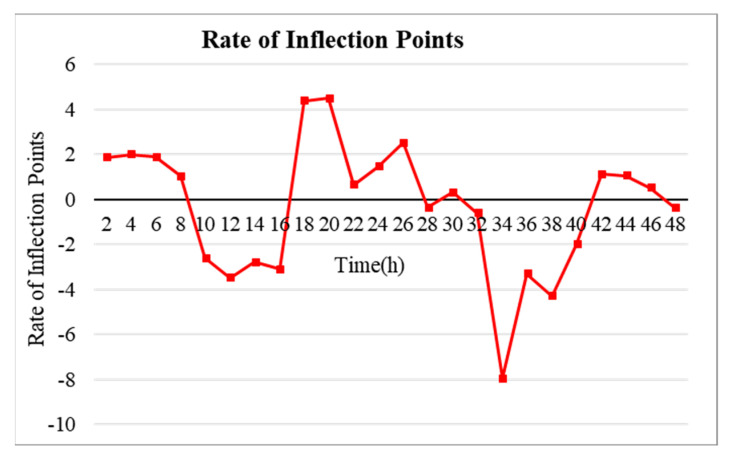
Rate of Inflection Points.

**Step 2:** Extracting The inflection point (Figure 6).

**Figure 7:**
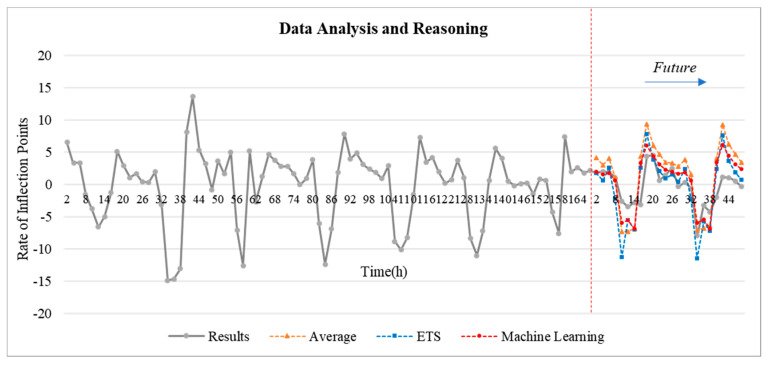
Data Analysis and Forecasting.

**Figure 8:**
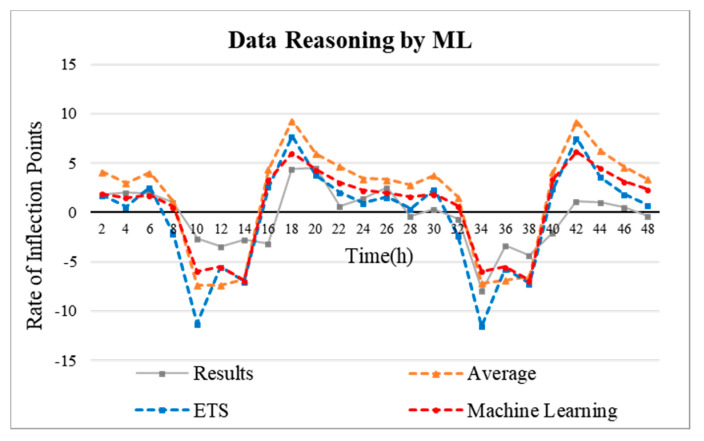
Data Forecasting and Reasoning by ML.

**Figure 9:**
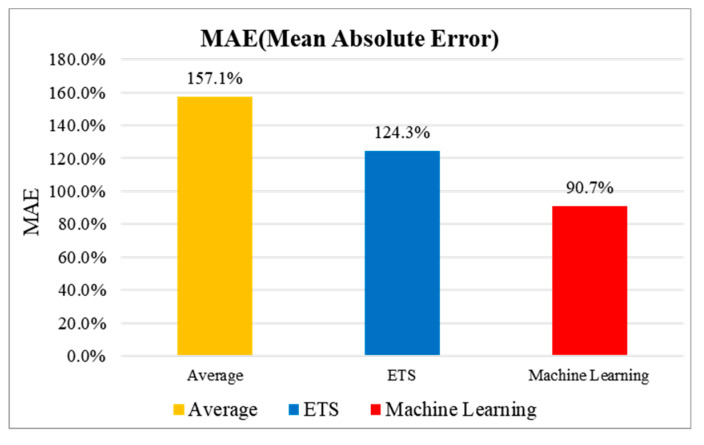
Error Rate by MAE (Mean Absolute Error) Method.

**Step 3:** After analyzing the temperature data of the hotel rooms for the past seven days, predicted after the change trend in the inflection point data was analyzed for two days (Figure 7, Figure 8 and Figure 9)

**Step 4:** Finally applied to the schedule setting of the HVAC system based on the predicted data and the past power (fan coil) data (Section 5.3).

Figure 5 shows a graph of the internal temperature change of the hotel. The data collection period is 25 November 2019 to 1 December 2019, and it was collected by comparing the room and outdoor temperatures in real time. Only the difference was analyzed for the temperature change trend in the room. The reason for measuring the difference between the indoor and outdoor temperatures is to analyze whether any event occurred in the room. In other words, it is to analyze the factors that caused the temperature difference in the room and to remove the change due to the external temperature. The difference between the internal and external temperatures shown in Figure 5 (orange) represents the temperature change (removal of the effect of external environmental factors) due to only the passive and active elements [2,45] that are inside the building. This allows for the determination of the events that have occurred inside the room. Table 5 is an inference of events that may occur in the room. Events that may occur:

In other words, a sharp increase in the temperature difference may be due to sunlight entering the room, and a slow increase may mean that the user manipulated the temperature on the HVAC system panel. In addition, a rapidly decreasing temperature difference means that the customer or manager has lowered the temperature by opening the window, and a slowly descending temperature difference indicates that the HVAC system was turned off. The main factor of the change in the difference temperature was confirmed that cannot escape from the four frameworks. Figure 6 shows the inflection points of the temperature difference change. This graph is obtained by differentiating the temperature data graph. That is, the inflection points were extracted from the difference between the outdoor and indoor room temperatures. The inflection points represent the occurrence of events at the vertices of the inflection point graph. Here, owing to the extraction of the inflection points, it is possible to extract the on/off history of the thermostats in users’ rooms based on the factors affecting the most temperature changes through a trend analysis of the inflection points. Through this analysis of the on/off history of the user’s thermostat, it is possible to predict the future HVAC use schedule.

### 5.2. Inflection Point Reasoning

In this study, the inflection point trend analysis of the temperature difference data over the past seven days in the “S” hotel, as shown in Figure 7 and Figure 8, is used to infer the data for the next two days. The simple mean, exponential smoothing (ETS), and ML-based Arima model (Microsoft Azure Machine Learning Studio) were applied for the inflection point analysis and reasoning. As shown in Figure 9, the error rate was measured based on the mean absolute error (MAE). Based on the temperature data from 25 November 2019 to 1 December 2019, the data for the next two days were inferred.

In this paper, temperature data are analyzed. Temperature data is in the form of a time series. Time series refers to the flow of data distributed over time. There are quantitative prediction methods based on time-series data include classical methods such as moving average, exponential smoothing, and disaggregation methods, and probabilistic methods such as Arima model and econometric model. In this paper, the classical model simple moving average and exponential smoothing (ETS) method are used, and the probabilistic model ML-based Arima model is applied. The simple moving average method infers future data by averaging past data through Excel. The exponential smoothing (ETS) model is also a classical reasoning model for time series and is used for time series analysis. Next, the most optimized algorithm for time series analysis is the Arima model. This model is built on the recently released Microsoft Azure Machine Learning Studio, which makes it possible to take advantage of this program. The data were inferred using these three methods, and as a result, an analysis was obtained that the Arima model was highly accurate (Figure 9). The *y*-axis of Figure 9 is MAE (mean absolute error). The purpose of use is to evaluate the numerical prediction model. The equation is as follows [46].

(1) $$MAE=\frac{1}{n}\sum \left|\hat{y}-y\right|$$

In simple terms, it is the average of the absolute values of (predicted value-actual value). In other words, it is the average of the absolute values of the errors. This allows you to compare the residuals between models. However, since MAE is the average of the error distance, when comparing models with large differences in the size of the error average, a model with a smaller error average is evaluated as a better model. Here, it is the most accurate at 0%, and the higher the value, the more inaccurate. The value can be over 100%.

Through this temperature change rate inference, it is possible to predict the future use of the HVAC system and thus set a schedule for future energy savings. This schedule is used to provide HVAC system control guidelines based on predicted data. What can be obtained due to the inference of the inflection point of the sensor data is that the user’s QoS reward can be extracted. That is to say, the user’s complaint is received by the user’s direct intervention from the point where the user has raised or lowered the temperature due to the transition of the inflection point. The following section shows how to save energy based on these user rewards.

### 5.3. Purpose-Oriented Optimization Method

The aim of the purpose-oriented HVAC system optimization is to achieve energy savings by setting energy saving goals in advance. It has been determined that it is impossible to reduce the total energy and measure the exact amount of energy savings in the various HVAC facilities of a BEMS. Therefore, this paper suggests a method considering an HVAC system (heating) that exhibits time dependent characteristics. The energy savings target (initial setting value) is assumed to be 25%. It is also assumed that energy savings in HVAC systems have time-dependent characteristics.

Time-dependent means that the energy consumption is almost proportionally increased over time, and an energy reduction of 25% means that the operation time of the HVAC system (boiler) is reduced by 25%. In other words, the energy consumption of the HVAC system is reduced by 25% when assuming a 25% time-dependent reduction in the uptime. An important aspect of this is determining how the operation of the HVAC system (boiler) can be reduced to achieve a 25% energy savings. This is based on the results of data analysis, shown in Figure 8. The operating policy of the HVAC system is regulated through the temperature data inflection point of the ML-based inferred point shown in Figure 8. That is to say, the HVAC system is controlled to find an optimized time domain for energy savings to reduce energy consumption in that time zone. From the inflection point inference graph presented in Figure 8, it is possible to classify the highest and lowest time zones of the inflection point. The point at which the temperature change increases is the highest inflection point, and the point at which the temperature change decreases can be said to be the lowest inflection point. This means that the inflection point is in ‘+’ time zone, this means that the temperature change increases, and the inflection point is in ‘−’ time zone, the temperature change is lowered. Thus, it is possible to deduce the following interpretations.

**Figure 10:**
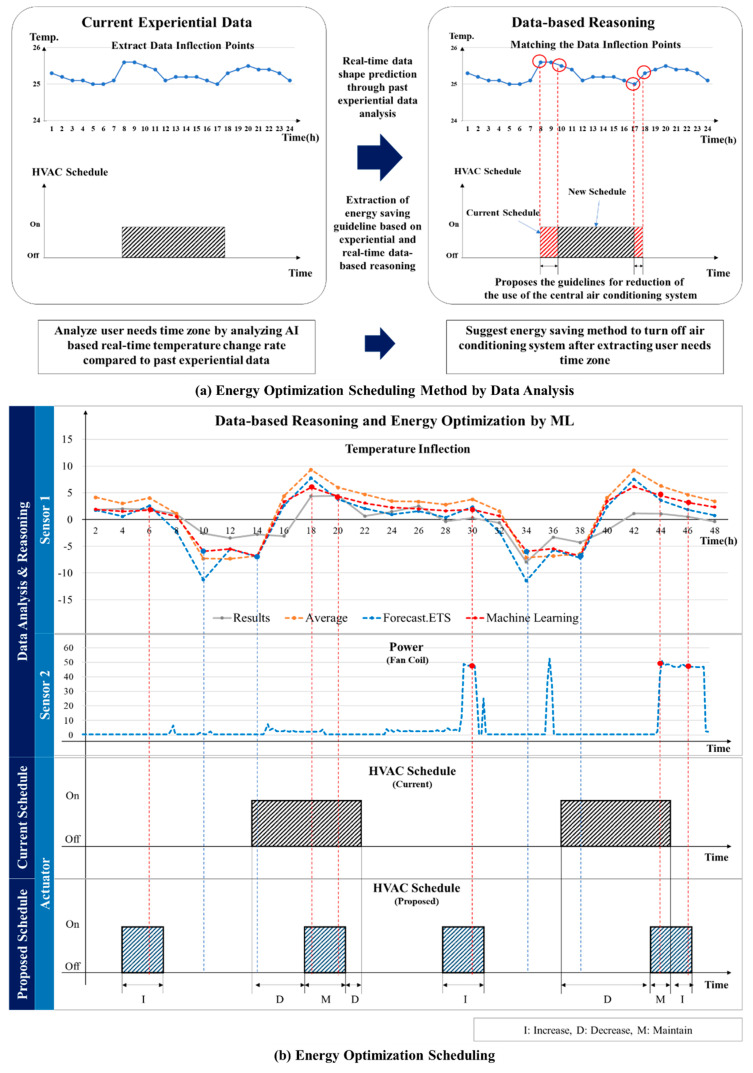
Energy Optimization Scheduling Method.

This study determined that there is no situation other than those presented in Table 6. The selection of the optimal energy saving time of the HVAC system through the inflection point data analysis results presented above suggests a method for achieving purpose-oriented energy savings, as shown in Figure 10. Based on the inferred data, the causes of the temperature increase and decrease inside the room can be said to be the user’s request and the influence of the external environment. Of these, the user’s request is the most important. Regardless of whether or not any change occurs owing to external factors (sunlight, air inflow due to ventilation), the purpose of the HVAC system is to determine the optimal point that may be requested by the user to increase the user’s QoS. That is to say, it is possible to control the optimization to achieve energy savings within the user’s satisfaction range by applying a schedule for which the HVAC system is activated when the inflection point is positive and not activated when the inflection point is negative. In addition, to measure the power of the room’s fan coil to introduce the hot air inside the room to increase the accuracy, the on/off state of the fan coil was measured. That is, the HVAC system is turned on/off in accordance with past pan coil operation. In other words, the system plan was applied by scheduling a point that satisfies one of the cases shown in Table 7. Figure 10 shows a description of the plan for scheduling a system based on the inferred data.

## 6. Energy Usage Optimization

### 6.1. HVAC Scheduling Optimization for Energy Saving

Figure 10 shows the optimal control of the HVAC system achieved via changing the HVAC system schedule. Usually, the existing building is the central HVAC system. To control this central HVAC system, it is important to establish a careful pre-schedule policy. Because the guest cannot control the room temperature directly, and owing to remnants of the cold heat generated by the HVAC, the customer may open the duct of the room at the required time to control the temperature inside the room. Therefore, it is important to supply the cold heat generated in the center by the HVAC system boiler by predicting the time zone at which it is required by the user. In this study, that time zone was determined by analyzing the temperature change inflection points collected from the user’s room in the past by time of day to operate the HVAC system optimally. The information from the inflection reasoning points in the analyzed temperature inflection point change graph provides energy saving scheduling guidelines.

It was confirmed that the central HVAC system (heating) in the hotel was operating in December for an average of 8–15 h (Operating History Report, 1 December 2018). To achieve a 25% energy reduction from the past eight hours of operation history, it was necessary to shorten the total operation time by two hours and save a total of 6–12 h of uptime. To decrease the operation by two hours compared to the eight hours for which the HVAC was operating in the past, it will be necessary to operate the HVAC system intensively in the sections of the temperature history with user requests. Table 8 shows the expected benefits of the proposed system.

As seen in Figure 10, the proposed system was applied to the scheduling of the HVAC system using the data inferred based on ML. By analyzing the inferred data inflection points, it was confirmed that the temperature change was greatest at the vertices of the inflection points, and the HVAC system schedule was adjusted near the inflection points. That is, based on the comparison with the past operation of the HVAC system, a dynamic scheduling is set up so that the HVAC system is operated at times corresponding to the vertices of the positive inflection points, and the operation of the HVAC system is reduced at times corresponding to the vertices of the negative inflection points. In addition, the fan coil data was also analyzed to operate the HVAC system primarily at the points where the fan coil was operated in the past. The resultant energy savings rate is estimated to be 20–25. It will continue to be optimized by the RL-based optimization method presented in Section 3 to achieve optimal energy savings between the user and the HVAC system.

### 6.2. RL-Based Algorithm

The following steps (Box 3) are required to obtain a typical RL algorithm [47].

Box 3 **Main procedure of RL algorithm.**

**Main procedure of RL algorithm**

**Step 1:** The agent interacts with the environment by performing an action.

**Step 2:** The agent performs an action and moves from one state to another.

**Step 3:** The agent will receive a reward based on the action it performed.

**Step 4:** The agent will understand whether the action was good or bad.

**Step 5:** If the agent received a positive reward, the agent will prefer performing that action or else the agent will try performing another action, which results in a positive reward. It is basically a trial and error learning process.

#### 6.2.1. Markov Decision Process (MDP)

The Markov decision process (MDP) provides a mathematical framework for modeling decision-making situations. Almost all the reinforcement learning problems can be modeled as a MDP [44]. The following important elements represent the MDP (Table 9):

As shown in Figure 1a, the agent receives status $s_{t}$ from the environment at every point in time, and selects the optimal action $a_{t}$ from the set of possible actions through its policy π. The environment moves to the new state $s_{t+1}$ according to the agent’s action and provides the agent with a reward $r_{t+1}$ for the action. The following shows return Equation (2), which represents the total sum of rewards Equations (3) and (4), and the discount factor Equation (5).

Rewards and returns:

(2) $$R_{\;t}=r_{t+1}+\;r_{t+2}+\;r_{t+3}+\ldots \;r_{T}$$

(3) $$R\_A_{\;t}=QoS_{t+1}+\;QoS_{t+2}+\;QoS_{t+3}+\ldots \;QoS_{T}\;(\mathrm{Agent}\;A)$$

(4) $$R\_B_{\;t}=ES_{t+1}+\;ES_{t+2}+\;ES_{t+3}+\ldots \;ES_{T}\;(\mathrm{Agent}\;B)$$

(5) $$R_{t}=r_{t+1}+\;\gamma r_{t+2}+\;\gamma^{2}r_{t+3}+\gamma^{3}r_{t+4}+\ldots =\overset{\infty }{\underset{k=0}{\sum }}\gamma^{k}r_{t+k+1}$$

Discount factor:

Reinforcement learning is conducted as an evaluation of the future reward $E_{\pi }\left[\;R_{t}|s_{t}=s\right]$, if the environment continues to act according to policy ‘π’. This is called the state value function Equations (6) and (7). It shows how good the resulting state ‘s’ is according to policy π.

State value function:

(6) $$V^{\pi }\left(s\right)=\;E_{\pi }\left[\;R_{t}|s_{t}=s\right]$$

(7) $$V^{\pi }\left(s\right)=\;E_{\pi }\left[\;\overset{\infty }{\underset{k=0}{\sum }}\gamma^{k}r_{t+k+1}\;|\;s_{t}=s\right]$$

The value considering the case of performing an action in a state is derived as a state-action value function (Q function). It is defined as $E_{\pi }\left[\;R_{t}|s_{t}=s,\;a_{t}=a,\;\right]$, the sum of the expected rewards when taking action a in state s and subsequently following a certain policy π. The purpose of the MDP is to find the policy with the greatest value through the state-action value function (Q Function). A state-action value function is also called the Q Function Equation (8). The Q Function specifies how good it is for an agent to perform a particular action in a state with a policy π [44].

State-action value function (Q function):

(8) $$Q\left(s,\;a\right)=\;E_{\pi }\left[\;\overset{\infty }{\underset{k=0}{\sum }}\gamma^{k}r_{t+k+1}\;|\;s_{t}=s,\;a_{t}=a\right]$$

To obtain the value function $V^{\pi }\left(s\right)$ considering the policy π, the expected value must be calculated for all future compensations, so the total sum of infinite future rewards at the current time t is obtained through the Bellman Equations (9) and (10) [44]. The Bellman equation is a method based on that the loop can be converged from the initial value to obtain the expected value of the infinite future. Expected values include the probability of doing something and the probability of going to a certain state when acting, so it can be defined the expected value through the policy and state variation probability in Bellman’s expectation equation. The value function starts by initializing to a meaningless initial value and converges so that the left and right terms have the same value through iterative calculation of the Bellman equation. The value function that maximizes future compensation is called the optimal value function, and the policy at this time means the optimal policy $\pi^{*}$, which has the highest expected value among all policies Equations (11) and (12).

#### 6.2.2. The Bellman Equation and Optimality

The Bellman equation is most important equation for solving the MDP. Solving the MDP means finding the optimal policies and value functions. There can be several different value functions according to different policies. The optimal value function $V^{*}\left(s\right)$ is the one that yields the maximum value compared with all other value functions [44]:

Bellman equation of optimal value function:

(9) $$V^{*}\left(s\right)=\;max_{\pi }V^{\pi }\left(s\right)$$

(10) $$V^{*}\left(s\right)=\;max_{a}Q^{*}\left(s,a\right)$$

Bellman equation of value and Q function:

(11) $$V^{\pi }\left(s\right)=\;\underset{a}{\sum }\pi \left(s,\;a\right)\underset{s^{\prime }}{\sum }P_{ss^{\prime }}^{a}\left[R_{ss^{\prime }}^{a}+\;\gamma V^{\pi }\left(s^{\prime }\right)\right]$$

(12) $$Q^{\pi }\left(s,\;a\right)=\;\underset{s^{\prime }}{\sum }P_{ss^{\prime }}^{a}\left[R_{ss^{\prime }}^{a}+\;\gamma \underset{a’}{\sum }Q^{\pi }\left(s^{\prime },\;a^{\prime }\right)\right]$$

(13) $$V^{*}\left(s\right)=\;max_{a}\underset{s^{\prime }}{\sum }P_{ss^{\prime }}^{a}\left[R_{ss^{\prime }}^{a}+\;\gamma \underset{a^{\prime }}{\sum }Q^{\pi }\left(s^{\prime },\;a^{\prime }\right)\right]$$

A Bellman equation can be driven for the Q function; the final equation is as follows:

(14) Q(s,a)=Transition Probability*(Reward Probability+Gamma*value_of_next_state)

**Figure 11:**
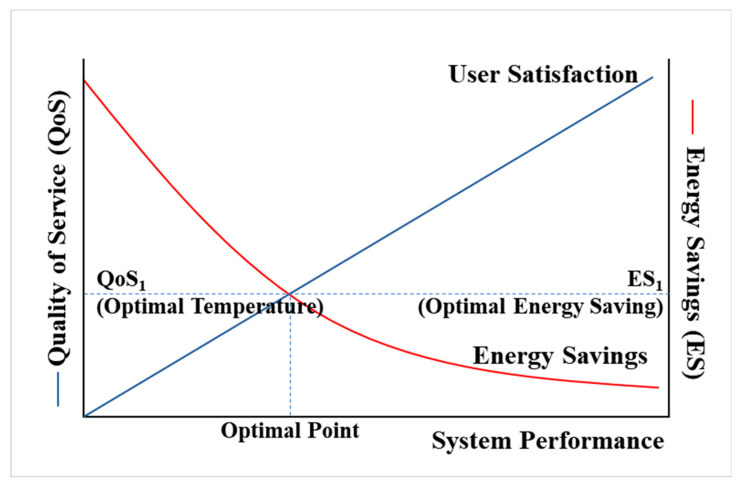
Relationship of QoS-ES-System Performance.

The optimal value function $V^{*}\left(s\right)$ is the sum of the rewards when following the optimal policy with the maximum value for all policies Equation (13). The optimal policy can be obtained by updating the current policy with a better policy. It is an optimal value function that is converted into a form that can calculate expected values. The optimal action value function can also be derived in the same way. Optimal policy means the action with the largest value of the optimal value function in each state. Through Figure 2 and Figure 11, we can find the best point to satisfy the user’s optimal QoS and save the proper energy, and search for the best point through RL-based BEMS to save energy, at the same time, maintain user satisfaction.

### 6.3. RL-Based Optimization

First, before implementing the algorithm, it is required the pre-analysis of the relationship between the user and the air conditioning system to apply RL presented in this paper. As can be seen in Figure 1b, BEMS considers two aspects: user and HVAC. That is, in order to implement the algorithm considering two aspects of Agent 1 (user) and Agent 2 (HVAC), two elements, Reward A and B must be required. As shown in Table 2, Reward A can be called User QoS. That is, the positive reward is set to the most optimal temperature, and the negative reward is unsatisfactory to the user. Reward B can be used as energy use, positive reward means that energy use decreases, negative reward means that energy use increases. In other words, as shown in Figure 11, you should find the best point to satisfy these two rewards at the same time without biasing them in one place. Figure 11 below shows a graph of QoS and energy saving (ES). It has two properties as follows [48].

1. It consumes unnecessary energy even though it already satisfies QoS
2. Energy is saved too much and QoS is not satisfied

As showed in Figure 11, it needs to find the optimal point to satisfy the user’s optimal QoS while saving the energy. Therefore, this research proposed a system architecture that can save energy by searching for the optimal point through RL-based BEMS and maintain user satisfaction.

First, as a Box 4, in order to perform RL-based optimization, the architecture suggested in Section 3 must be linked with BEMS. In other words, there must be an analysis of what Agent, Environment, Action, State, and Reward are in BEMS. The Table 10 shows the analysis.

Box 4 **RL-based optimization procedure.**

**RL-based optimization procedure**

***1. User-BEMS side***

**Step 1:** Action A: Agent A (Guest) will raise and lower the temperature to satisfy the temperature. This was called Action A.

**Step 2:** State A: Accordingly, the temperature inside the room would be changed, and the temperature inside the room at this time was referred to as State A (*T*_1_ (21 °C) to *T*_5_ (25 °C)).

**Step 3:** Reward A: At this time, the reward that the user can receive can be said to be user satisfaction (−1 and 1).

***2. BEMS-HVAC system side***

**Step 4:** Action B: Agent B (BEMS) will turn HVAC on/off to maintain temperature and save energy. This was called Action B.

**Step 5:** State B: Accordingly, the heating system will repeatedly turn on/off and change the temperature inside the building, and the state of energy use at this time is called State B (P_1_ (21%) to P_5_ (25%)).

**Step 6:** At this time, the Reward that the BEMS can receive may be referred to as an Energy Saving Grade (SAG). Energy efficiency ratings are given in the form of rewards with a decimal value between (−1 and 1).

As can be seen in the Table 10, BEMS can be viewed from two aspects as shown in Figure 1. If the relationship between Guest and BEMS is A, and the relationship between BEMS and HVAC is B, Agent A can be considered as Guest. Guest will raise and lower the temperature to satisfy the temperature. This was called Action A (Step 1). Accordingly, the temperature inside the room would be changed, and the temperature inside the room at this time was referred to as State A (Step 2). State A is assumed to range from *T*_1_ (21 °C) to *T*_5_ (25 °C) as the temperature state. At this time, the reward that the user can receive can be said to be user satisfaction. User satisfaction is given in the form of a reward with a decimal point value between (−1 and 1) (Step 3). On the other hand, Agent B can be viewed as BEMS. BEMS will turn HVAC on/off to maintain temperature and save energy. This was called Action B (Step 4). Accordingly, the heating system will repeatedly turn on/off and change the temperature inside the building, and the state of energy use at this time is called State B (Step 5). State B is Energy Saving State (ES), which is assumed to range from P_1_ (21%) to P_5_ (25%). At this time, the Reward that the BEMS can receive may be referred to as an energy saving grade (SAG). Energy efficiency ratings are given in the form of rewards with a decimal value between (−1 and 1) (Step 6).

The important part here is how to extract the rewards from A and B. In order to calculate user satisfaction and energy efficiency class, this paper approached the inflection point point presented above. As shown in Table 6, when the inflection point is a + part and a − part, Table 11 shows the reward relationship can be extracted.

**Figure 12:**
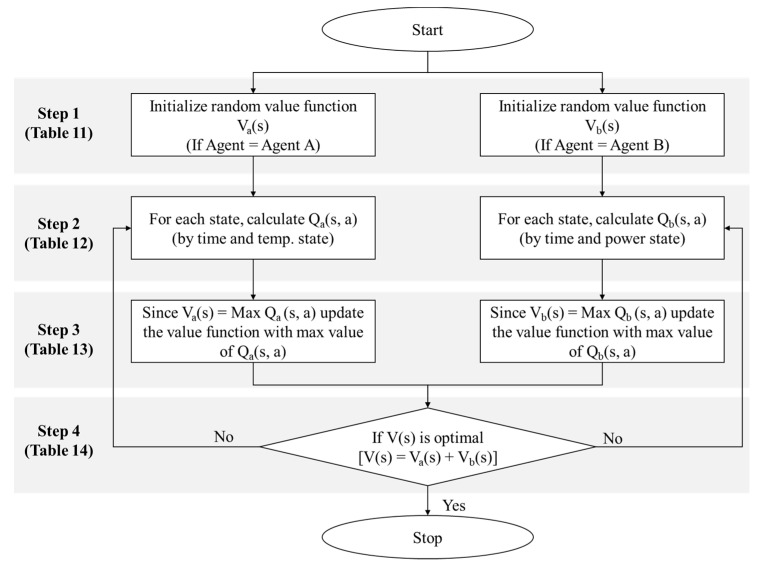
Value iteration algorithm with the Bellman equation for RL-based BEMS optimization by MDP.

Figure 12 and Box 5 shows the revised value iteration algorithm by Bellman equation in dynamic programming for RL-based BEMS [47]. Value iteration algorithm of the Bellman equation produces representative algorithm for solving problems about Markov decision process (MDP) [44,47,49,50]. Reinforcement learning is a theory that emerged to solve the MDP problem, and it is a learning method that allows agents under the state to select the optimal action through interaction with the environment. It is done similarly to the process of learning new knowledge by a person, and by increasing the good behavior more and more by differentiating the reward according to the behavior, the agent can learn even without any prior knowledge. This indicates the RL-based algorithm formula presented in this paper. Next, we explain the step-by-step optimization via the value iteration algorithm.

Box 5 **Value iteration algorithm for RL-based optimization.**

**Value iteration algorithm for RL-based optimization**

**Step 1:** Initialize the random value function of A and B (Table 12)

**Step 2:** For each state, calculate Q_a_ (s, a) and Q_b_ (s, a): Q table (Table 13 and Table 14)

**Step 3:** Update the value function with the max value of Q_a_ (s, a) and Q_b_ (s, a): Q Function table (Table 15)

**Step 4:** Estimated optimal value function table (Table 16)

### 6.4. Implementation

**Figure 13:**
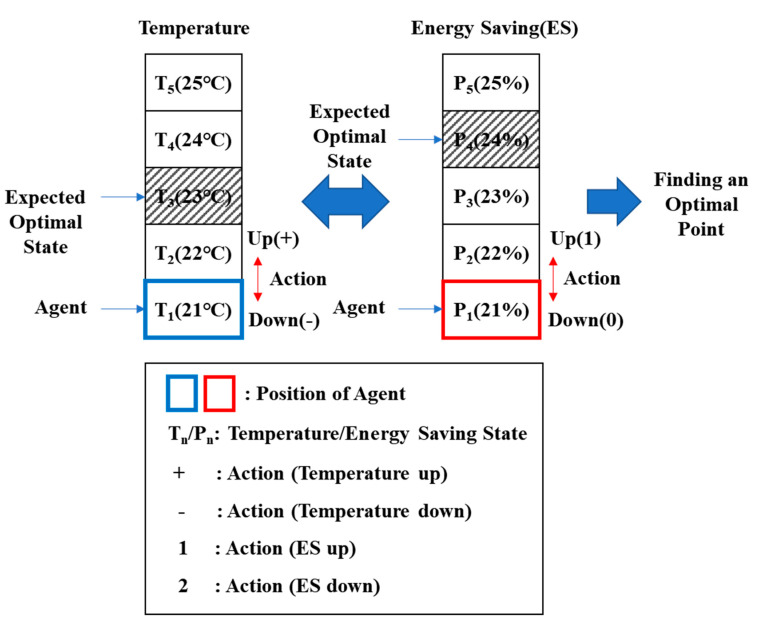
The method of finding an optimal point.

Figure 13 is a diagram of the RL-based optimization process. The temperature and energy-saving areas were divided into five zones each. Each zone represents a state, which in turn represents the temperature and energy-saving rate allocated from *T*_1_ to *T*_5_ and from P_1_ to P_5_, respectively. Agents in each area perform an action (up/down) aimed at finding the most optimal temperature and energy-saving rates. The most important aspect here is to find the optimum value based on the previous temperature data. A step-by-step explanation is presented next. The algorithm shown in Figure 12 is used to find the optimal value. Using this algorithm, the following initial random value function, and the transition and reward probabilities were extracted. Table 12 shows the initial random value function of RL-based BEMS [47].

#### 6.4.1. Step 1: Initialize Random Value Function of A and B

Table 12 is called an initial random value function table. To initialize the table, all states assigned zeros, as shown.

#### 6.4.2. Step 2: For Each State, Calculate *Q_a_* (*s*, *a*) and *Q_b_* (*s*, *a*): *Q* Table

Table 13 shows the transition and reward probabilities used to obtain the *Q* table. The *Q* value implies the value of an action in each state. For calculating (11), the transition and reward probabilities are needed. The transition ($P_{ss^{\prime }}^{a})$ and reward ($R_{ss^{\prime }}^{a}$) probabilities are shown as following:

It is worth noting that the transition ($P_{ss^{\prime }}^{a})$ and reward ($R_{ss^{\prime }}^{a}$) probabilities are calculated based on the user’s QoS and energy efficiency rating scenarios, which reflects data from the previous seven days. It is crucial to find the optimal point following this procedure because it is measured based on the user’s preferred scenarios. The *Q* Function table can be obtained following Table 13 step-by-step and the calculation Equation (3). The following is a detailed description of this process.

As suggested in Section 6.2.1, the *Q* Function can be obtained as the following equation [47].

Q(s,a)=Transition Probability*(Reward Probability+Gamma*value_of_next_state)

Gamma is the discount factor; it was set as 0.6.

*Q* value for state $T_{1}$ and Action +:

(15) $$\begin{array}{ll} Q\left(T_{1},\;+\right)\;=\;P_{T_{1}T_{1}}^{+} & *\left(R_{T_{1}T_{1}}^{+}+\;\gamma \;*Value\;of\;T_{1}\right)+\;P_{T_{1}T_{2}}^{+}*\left(R_{T_{1}T_{2}}^{+}+\;\gamma \;*Value\;of\;T_{2}\right)\\ & +\;P_{T_{1}T_{3}}^{+}*\left(R_{T_{1}T_{3}}^{+}+\;\gamma \;*Value\;of\;T_{3}\right)+P_{T_{1}T_{4}}^{+}\\ & *\left(R_{T_{1}T_{4}}^{+}+\;\gamma \;*Value\;of\;T_{4}\right)+P_{T_{1}T_{5}}^{+}*\left(R_{T_{1}T_{5}}^{+}+\;\gamma \;*Value\;of\;T_{5}\right)\\ & =0.96 \end{array}$$

*Q* value for state $T_{1}$ and Action −:

(16) $$\begin{array}{ll} Q\left(T_{1},\;-\right)\;=\;P_{T_{1}T_{1}}^{-} & *\left(R_{T_{1}T_{1}}^{-}+\;\gamma \;*Value\;of\;T_{1}\right)+\;P_{T_{1}T_{2}}^{+}*\left(R_{T_{1}T_{2}}^{-}-\;\gamma \;*Value\;of\;T_{2}\right)\\ & +\;P_{T_{1}T_{3}}^{-}*\left(R_{T_{1}T_{3}}^{-}+\;\gamma \;*Value\;of\;T_{3}\right)+P_{T_{1}T_{4}}^{-}\\ & *\left(R_{T_{1}T_{4}}^{-}+\;\gamma \;*Value\;of\;T_{4}\right)+P_{T_{1}T_{5}}^{+}*\left(R_{T_{1}T_{5}}^{-}+\;\gamma \;*Value\;of\;T_{5}\right)\\ & =0.06 \end{array}$$

*Q* value for state $P_{1}$ and Action 1:

(17) $$\begin{array}{ll} \;\;Q\left(P_{1},\;1\right)\;=\;P_{P_{1}P_{1}}^{1} & *\left(R_{P_{1}P_{1}}^{1}+\;\gamma \;*Value\;of{\;P}_{1}\right)+\;P_{P_{1}P_{2}}^{1}*\left(R_{P_{1}P_{2}}^{1}+\;\gamma \;*Value\;of{\;P}_{2}\right)\\ & +\;P_{P_{1}P_{3}}^{1}*\left(R_{P_{1}P_{3}}^{1}+\;\gamma \;*Value\;of{\;P}_{3}\right)+P_{P_{1}P_{4}}^{1}\\ & *\left(R_{P_{1}P_{4}}^{1}+\;\gamma \;*Value\;of{\;P}_{4}\right)+P_{P_{1}P_{5}}^{1}*\left(R_{P_{1}P_{5}}^{1}+\;\gamma \;*Value\;of{\;P}_{5}\right)\\ & =0.7 \end{array}$$

*Q* value for state $P_{1}$ and Action 0:

(18) $$\begin{array}{ll} \;\;Q\left(P_{1},\;0\right)\;=\;P_{P_{1}P_{1}}^{0} & *\left(R_{P_{1}P_{1}}^{0}+\;\gamma \;*Value\;of{\;P}_{1}\right)+\;P_{P_{1}P_{2}}^{0}*\left(R_{P_{1}P_{2}}^{0}+\;\gamma \;*Value\;of{\;P}_{2}\right)\\ & +\;P_{P_{1}P_{3}}^{0}*\left(R_{P_{1}P_{3}}^{0}+\;\gamma \;*Value\;of{\;P}_{3}\right)+P_{P_{1}P_{4}}^{0}\\ & *\left(R_{P_{1}P_{4}}^{0}+\;\gamma \;*Value\;of{\;P}_{4}\right)+P_{P_{1}P_{5}}^{0}*\left(R_{P_{1}P_{5}}^{0}+\;\gamma \;*Value\;of{\;P}_{5}\right)\\ & =-0.05 \end{array}$$

Next, these are updated in the *Q* Function table of the first iteration, as shown in Table 14.

This step must be repeated for several iterations, i.e., we repeat steps 2 (Table 15) and 3 (Table 16), while calculating the *Q* value and updating the value function until the optimal value is obtained. The *Q* Function tables of the second and third iterations are given in Table 15 and Table 16, respectively.

#### 6.4.3. Step 3: Update the Value Function with the Max Value of *Q_a_*(*s*, *a*) and *Q_b_*(*s*, *a*): *Q* Function Table

The Q Function can be calculated as Table 17 by applying the transition and reward probability in each state to obtain *Q* (*s*, *a*) to the Bellman Equation (14). This is the result of iteratively calculating the loop to minimize the change in the difference between each value in order to calculate the optimal value function. As a result, the estimated optimal value function as shown in the table was calculated.

#### 6.4.4. Step 4: Estimated Optimal Value Function Table

**Figure 14:**
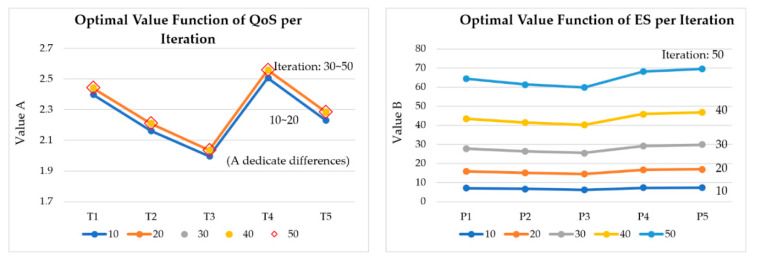
Estimated optimal values of QoS and energy saving per iteration.

**Figure 15:**
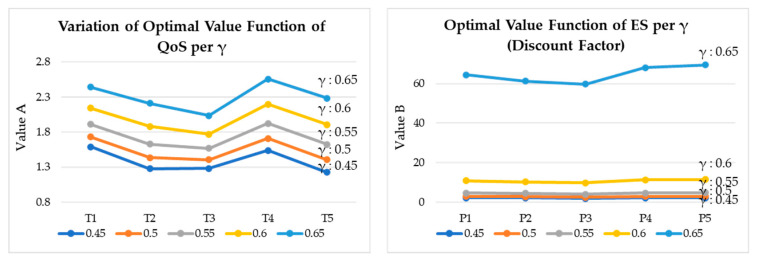
Estimated optimal values of QoS and energy saving per γ (Discount Factor).

**Figure 16:**
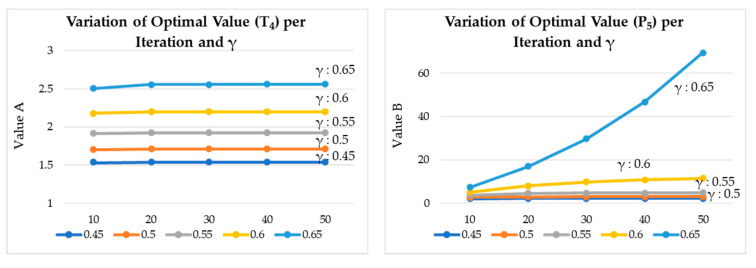
Variation of optimal values of QoS and energy saving per iteration and γ.

From this *Q* Function table, the maximal value is selected in each state. Finally, the optimal value function table is extracted, as shown below:

Figure 14 shows the estimated optimal values of QoS and energy saving per iteration. Figure 15 shows the estimated optimal values of QoS and energy saving per γ (discount factor). Figure 16 shows the variation of optimal values of QoS and energy saving per iteration and γ. Each graph represents a graph of changes according to the iteration of the algorithm and the change of the discount factor (γ). As the algorithm was iterated, a graph of a certain shape was drawn, and according to the change of γ, it was found that it was most stable when γ: 0.6.

Generally, high QoS and energy efficiency points are extracted at *T*_4_ (24 °C), *T*_5_ (25 °C), *T*_1_ (21 °C) and P_5_ (25%), P_4_ (24%), P_1_ (21%) based on the estimated optimal value function table as Table 18. It can be seen that when the RL-based simulation is implemented based on the past data, the point of the temperature and energy saving rate are generally optimized at the *T*_4_ (24 °C) and P_5_ (25%). However, this method is not yet a perfect optimization method. This is because the associated value between QoS and ES is not expressed. Currently, the RL-based energy optimization method has not been released yet, but it is necessary to continuously study how to provide the user’s QoS in this way while achieving optimal energy saving, and this method still requires a lot of research results. We expect to see an infinite prospect in the field of BEMS energy optimization.

## 7. Future Prospects and Business Model (BM)

**Figure 17:**
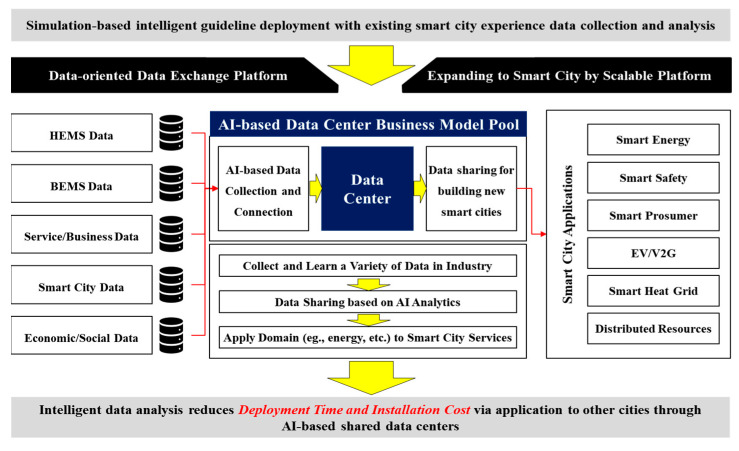
Business Model (BM) and Derivative Gains of the Proposed System.

This paragraph presents a business model (BM) that employs the BEMS presented in this paper (Figure 17). This paper has presented an RL-based purpose-oriented BEMS for energy usage optimization. The primary aim of this paper is to provide guidelines for reducing the energy usage of HVACs and simultaneously maintaining the users’ QoS via a BEMS. The proposed system will save 20–25% of the heating energy in the building. Importantly, these services are based on data that already exist. The advancement of the service is determined by the presence of data collected in the past. In this regard, the business model (BM) that can be created based on the system presented above provides energy saving guidelines through a data center. As shown in Figure 10 and Table 15, intelligent data analyses can reduce “deployment time and installation cost” via their application to other cities through AI-based shared data centers.

The return on investment (ROI) is extracted from BM 2 and BM 3, as shown in Table 19. In BM 2, the ROI per building was calculated to be approximately 7.14 years (if the energy saving rate is 25%, the hotel’s thermal energy savings is approximately $35,338, and the smart-IoT system installation costs are approximately $252,419), and in BM 2, the ROI was calculated to be 7.16 years [27], considering that the additional cost saving rate of the IoT system deployment is 15.9% [4,27]. By utilizing existing BEMS data and linking it to other regions, it is expected that RL-based data center-based services can create a new level of service based on the data from BEMSs, and the advanced service will play an important role in future ICT fields.

## 8. Conclusions

In this paper, an RL-based purpose-oriented BEMS for energy usage optimization has been presented. This study implemented smart IoT sensors in an actual hotel testbed. Currently, temperature, humidity, motion detection, and power data are transmitted from the room in real time, and it is possible to monitor and simulate the data in the data center. This will play an important role in expanding to the future smart cities and will serve as an innovative guideline for the intelligent deployment of IoT systems. RL in artificial intelligence plays a key role in applying intelligent elements in the field of ICT, and this paper uses the RL algorithm to implement and simulate a BEMS. This system established a structure for the distribution of rewards among users and the HVAC system through user–HVAC system interactions. Furthermore, an optimization method that achieves energy savings for the HVAC system while increasing the QoS through RL-based user-HVAC system interactions was proposed. In addition, using the ML-based temperature data inference and purpose-oriented energy reduction measures proposed in this study, it was possible to calculate a 25% energy saving expectation for the hotel’s HVAC system, which could substantially contribute to the technological advancement of intelligent BEMSs in the future.

## Figures and Tables

**Table 1:** Merits of the proposed system.

Existing System	Proposed System
Data prediction is performed via simple data analysis and energy optimization Energy saving is achieved by simple facility replacement of stand-alone systems after construction No purpose-oriented saving method	Reinforcement learning (RL)-based energy optimization IoT-based lightweight & cost-effective system for existing buildings Purpose-oriented saving method

**Table 2:** Relationship between the Agent and Environment in the BEMS.

Classification	A	B
Agent	All users: Guests	BEMS: IoT Infrastructure
Environment	BEMS: IoT Infrastructure	HVAC
Action	Setting the temperature: rise/down	Energy saving: HVAC on/off
State	Temperature state	Energy state
Reward	Positive reward: Positive QoS, Optimal temperature Negative reward: Negative QoS, Guest dissatisfaction	Positive reward: Energy saving Negative reward: Energy consumption
Final Purpose	Optimal system management for energy efficiency and user QoS	

**Table 3:** Hotel pre-analysis and smart-IoT installation.

Building Appearance	Characteristics
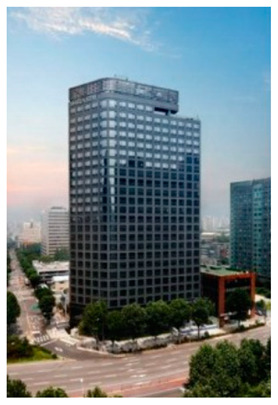	**Building structure** Total number of floors: 26 (floors 1 and 2: commercial rooms, 3–26: guest rooms) Total number of rooms: 383 Heating system: 2 (“A” heating system: Room 1 to Room 9; “B” heating system: Room 10 to Room 18 of the entire floor control) The heating system is designed to operate 24 h a day, and when guests check out, the cleaning personnel rely on manual ventilation, such as opening the windows to save energy. **Energy management type** Managed with 190 zones in the building **Individual room control method** Room air conditioning management is performed via a fan coil by the customer through the room temperature control unit.
**Smart-IoT Installation Site**	
**26 Floor—Total 4 Rooms**	**25 Floor—Total 4 Rooms**
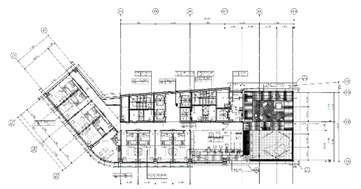	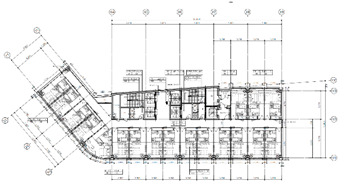
**15 Floor—Total 8 Room**	**3 Floor—Total 8 Room**
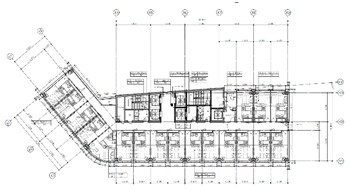	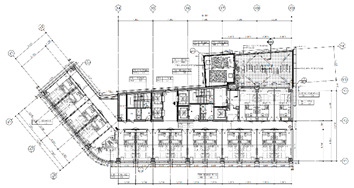

**Table 4:** Specifications of installed sensors.

Items		Characteristics	Uses
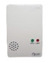	Temperature and humidity sensor	Type: Sensor Operation range: −55 °C–200 °C Communication: Zigbee	Collecting indoor temperature/humidity data
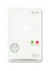	Smart fine dust/CO2 sensor	Measurement items: Carbon monoxide, Carbon dioxide, Methane, Formaldehyde, VOCs, Particles	Collecting indoor CO_2_, fine dust data
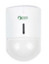	Smart motion sensor	Detection distance: 10 m/84° Sensing space range: 5.0 × 5.0 × 5.0 (m)	Indoor user movement detection
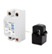	Smart submeter	Type: Sensor Size: 7.5 × 7.5 × 3.5 (cm) Communication: Zigbee	Measure the room’s power utilization

**Table 5:** Expected Events in the Room by Analyzing the Temperature Graph.

	Rapidly	Slowly
**Rising**	Sunlight shines into the room	HVAC system on
**Descending**	Ventilation due to windows	HVAC system off

**Table 6:** Interpretations and Reasonings of Inflection Points.

	Inflection Point: +	Inflection Point: −
Meaning	Temperature change increases	Temperature change decreases
Reasoning 1	The time in which the user is not satisfied with the current temperature and raises the temperature through the temperature control panel, or the interval in which the user requests a temperature increase to the administrator.	The time in which the user is very satisfied with the current temperature and lowers the temperature through the temperature control panel, or the interval in which the user requests a temperature descent to the administrator.
Reasoning 2	Sunlight causes an increase room temperature.	The user opens the porch and windows to lower the temperature or proceeds with ventilation.
Reasoning 3	The number of users increases, thereby increasing the room temperature owing to the users’ body temperatures (It seems that this is almost impossible because the number of people allowed in the rooms is limited).	If the administrator turns the HVAC system off or on for cleaning, the windows are also opened and ventilation occurs.

**Table 7:** HVAC System Scheduling Point Based on Inferred Data.

	HVAC System On	HVAC System Off
**Case 1: Temperature Data**	Point of positive inflection	Point of negative inflection
**Case 2: Power Data** (**Fan Coil**)	Point of fan coil operation	Point of non-operation of fan coil

**Table 8:** Expected Benefits of the Proposed System.

Classification	Operation Time	Time Zone	Expected Energy Saving Rate
Current System	8	Unassigned	-
Proposed System	6	AM 3 h, PM 3 h	20–25%

**Table 9:** Elements description of MDP.

Element	Description
*S*	A set of states (*s*) the agent can actually be in.
*A*	A set of actions (*a*) that can be performed by an agent for moving from one state to another.
γ	Discount factor, which controls the importance of immediate and future rewards.
$P_{ss^{\prime }}^{a}$	Transition probability, which is the probability of moving from one state (*s*) to another (*s’*) by performing some action *a*.
$R_{ss^{\prime }}^{a}$	Reward probability, which is the probability of an agent acquiring a reward for moving from one state (*s*) to another (*s’*) by performing some action *a.*

**Table 10:** The factor of RL-based BEMS.

Classification	A	B
Agent	Guests	BEMS
Environment	BEMS	HVAC
Action	Temperature rise (+)/down (−)	HVAC on (1)/off (0)
State	Temperature state (*T*_1_~*T*_5_ °C)	Energy saving state (P_1_~P_5_%)
Reward	Positive user QoS/Negative user QoS (−1~1)	Energy saving/Energy consumption (−1~1)

**Table 11:** Relationship of reward and inflection point.

Classification	Inflection Point: +	Inflection Point: −
Reward A	Negative Reward: Negative QoS (−1~0)	Positive Reward: Positive QoS (0~1)
Reward B	Positive Reward: Positive ES (0~1)	Negative Reward: Negative ESG (−1~0)

**Table 12:** Initial random value function of RL-based BEMS.

State A (Temperature)	Value A	State B (Energy Saving)	Value B
*T*_1_ (21 °C)	0	P_1_ (21%)	0
*T*_2_ (22 °C)	0	P_2_ (22%)	0
*T*_3_ (23 °C)	0	P_3_ (23%)	0
*T*_4_ (24 °C)	0	P_4_ (24%)	0
*T*_5_ (25 °C)	0	P_5_ (25%)	0

**Table 13:** The transition and reward probability of RL-based BEMS.

State	Action	Next State	$P_{ss^{\prime }}^{a}$	$R_{ss^{\prime }}^{a}$	State	Action	Next State	$P_{ss^{\prime }}^{a}$	$R_{ss^{\prime }}^{a}$
*T*_1_	+	*T*_1_	0.3	1	P_1_	1	P_1_	0.2	0.8
*T*_1_	+	*T*_2_	0.4	1	P_1_	1	P_2_	0.5	0.6
*T*_1_	+	*T*_3_	0.2	1	P_1_	1	P_3_	0.4	0.4
*T*_1_	+	*T*_4_	0.1	0.6	P_1_	1	P_4_	0.3	0.2
*T*_1_	+	*T*_5_	0	0.7	P_1_	1	P_5_	0.2	0.1
*T*_1_	-	*T*_1_	0.3	0.2	P_1_	0	P_1_	0.3	0.1
*T*_1_	-	*T*_2_	0	−0.2	P_1_	0	P_2_	0.2	−0.2
*T*_1_	-	*T*_3_	0	−0.2	P_1_	0	P_3_	0.1	−0.4
*T*_1_	-	*T*_4_	0	−0.2	P_1_	0	P_4_	0	−0.6
*T*_1_	-	*T*_5_	0	−0.3	P_1_	0	P_5_	0	−0.8
*T*_2_	+	*T*_1_	0.1	−0.1	P_2_	1	P_1_	0.1	1
*T*_2_	+	*T*_2_	0.3	0.2	P_2_	1	P_2_	0.2	0.8
*T*_2_	+	*T*_3_	0.4	1	P_2_	1	P_3_	0.5	0.6
*T*_2_	+	*T*_4_	0.2	0.5	P_2_	1	P_4_	0.4	0.4
*T*_2_	+	*T*_5_	0.1	0.6	P_2_	1	P_5_	0.3	0.2
*T*_2_	-	*T*_1_	0.4	1	P_2_	0	P_1_	0.5	0.3
*T*_2_	-	*T*_2_	0.3	0.2	P_2_	0	P_2_	0.3	0.1
*T*_2_	-	*T*_3_	0.1	−0.2	P_2_	0	P_3_	0.2	−0.2
*T*_2_	-	*T*_4_	0	−0.2	P_2_	0	P_4_	0.1	−0.4
*T*_2_	-	*T*_5_	0	−0.2	P_2_	0	P_5_	0	−0.6
*T*_3_	+	*T*_1_	0	−0.2	P_3_	1	P_1_	0	1
*T*_3_	+	*T*_2_	0	−0.1	P_3_	1	P_2_	0.1	1
*T*_3_	+	*T*_3_	0.2	0.2	P_3_	1	P_3_	0.2	0.8
*T*_3_	+	*T*_4_	0.3	1	P _3_	1	P_4_	0.5	0.6
*T*_3_	+	*T*_5_	0.2	1	P _3_	1	P_5_	0.4	0.4
*T*_3_	-	*T*_1_	0.3	0.4	P_3_	0	P_1_	0.4	0.5
*T*_3_	-	*T*_2_	0.4	1	P_3_	0	P_2_	0.5	0.3
*T*_3_	-	*T*_3_	0.2	1	P_3_	0	P_3_	0.3	0.1
*T*_3_	-	*T*_4_	0	−0.2	P_3_	0	P_4_	0.2	−0.2
*T*_3_	-	*T*_5_	0	−0.2	P_3_	0	P_5_	0.1	−0.4
*T*_4_	+	*T*_1_	0	−0.3	P_4_	1	P_1_	0	1
*T*_4_	+	*T*_2_	0	−0.2	P_4_	1	P_2_	0	1
*T*_4_	+	*T*_3_	0	−0.1	P_4_	1	P_3_	0.1	1
*T*_4_	+	*T*_4_	0.2	1	P_4_	1	P_4_	0.2	0.8
*T*_4_	+	*T*_5_	0.3	0.4	P_4_	1	P_5_	0.5	0.6
*T*_4_	-	*T*_1_	0.2	0.5	P_4_	0	P_1_	0.3	0.7
*T*_4_	-	*T*_2_	0.3	0.4	P_4_	0	P_2_	0.4	0.5
*T*_4_	-	*T*_3_	0.4	1	P_4_	0	P_3_	0.5	0.3
*T*_4_	-	*T*_4_	0.2	1	P_4_	0	P_4_	0.3	0.1
*T*_4_	-	*T*_5_	0.1	−0.2	P_4_	0	P_5_	0.2	−0.2
*T*_5_	+	*T*_1_	0	−0.4	P_5_	1	P_1_	0	1
*T*_5_	+	*T*_2_	0	−0.3	P_5_	1	P_2_	0	1
*T*_5_	+	*T*_3_	0	−0.2	P_5_	1	P_3_	0	1
*T*_5_	+	*T*_4_	0	−0.1	P_5_	1	P_4_	0.1	1
*T*_5_	+	*T*_5_	0.2	1	P_5_	1	P_5_	0.2	0.8
*T*_5_	-	*T*_1_	0.1	0.6	P_5_	0	P_1_	0.2	0.9
*T*_5_	-	*T*_2_	0.2	0.5	P_5_	0	P_2_	0.3	0.7
*T*_5_	-	*T*_3_	0.3	0.4	P_5_	0	P_3_	0.4	0.5
*T*_5_	-	*T*_4_	0.4	0.3	P_5_	0	P_4_	0.5	0.3
*T*_5_	-	*T*_5_	0.2	1	P_5_	0	P_5_	0.3	0.1

**Table 14:** Q Function table of the first iteration.

State A	Action A	Value A	State B	Action B	Value B
*T*_1_	+	0.96	P_1_	1	0.7
*T*_1_	−	0.06	P_1_	0	−0.05
*T*_2_	+	0.61	P_2_	1	0.78
*T*_2_	−	0.61	P_2_	0	0.1
*T*_3_	+	0.54	P_3_	1	0.72
*T*_3_	−	0.72	P_3_	0	0.3
*T*_4_	+	0.32	P_4_	1	0.56
*T*_4_	−	0.8	P_4_	0	0.55
*T*_5_	+	0.2	P_5_	1	0.26
*T*_5_	−	0.6	P_5_	0	0.77

**Table 15:** Q Function table of the second iteration.

State A	Action A	Value A	State B	Action B	Value B
*T*_1_	+	1.4136	P_1_	1	1.384
*T*_1_	−	0.2328	P_1_	0	0.2128
*T*_2_	+	1.0822	P_2_	1	1.4046
*T*_2_	−	0.8234	P_2_	0	0.5704
*T*_3_	+	0.8424	P_3_	1	1.206
*T*_3_	−	1.1256	P_3_	0	0.945
*T*_4_	+	0.524	P_4_	1	0.9014
*T*_4_	−	1.3298	P_4_	0	1.2724
*T*_5_	+	0.272	P_5_	1	0.386
*T*_5_	−	1.0044	P_5_	0	1.3238

**Table 16:** Q Function table of the third iteration.

State A	Action A	Value A	State B	Action B	Value B
*T*_1_	+	1.689036	P_1_	1	1.964788
*T*_1_	−	0.314448	P_1_	0	0.440032
*T*_2_	+	1.379596	P_2_	1	1.937052
*T*_2_	−	1.041596	P_2_	0	0.989092
*T*_3_	+	1.034964	P_3_	1	1.648428
*T*_3_	−	1.369248	P_3_	0	1.502736
*T*_4_	+	0.660368	P_4_	1	1.182188
*T*_4_	−	1.654412	P_4_	0	1.885912
*T*_5_	+	0.320528	P_5_	1	0.4952
*T*_5_	−	1.336968	P_5_	0	1.948352

**Table 17:** *Q* Function table with max iteration value.

State A	Action A	Value A	State B	Action B	Value B
*T*_1_	+	2.140461707	P_1_	1	10.83261728
*T*_1_	−	0.445283107	P_1_	0	3.714355894
*T*_2_	+	1.879474769	P_2_	1	10.35792026
*T*_2_	−	1.398131522	P_2_	0	7.037297157
*T*_3_	+	1.376917547	P_3_	1	8.644272198
*T*_3_	−	1.768587559	P_3_	0	9.777649313
*T*_4_	+	0.927345189	P_4_	1	5.946635124
*T*_4_	−	2.197902471	P_4_	0	11.29521464
*T*_5_	+	0.429064595	P_5_	1	2.315948923
*T*_5_	−	1.908871623	P_5_	0	11.55501165

**Table 18:** Estimated optimal value function table (γ: 0.6).

State A	Value A	State B	Value B
*T*_1_ (21 °C)	2.1404617	P_1_ (21%)	10.832617
*T*_2_ (22 °C)	1.8794748	P_2_ (22%)	10.35792
*T*_3_ (23 °C)	1.7685876	P_3_ (23%)	9.7776493
*T*_4_ (24 °C)	2.1979025	P_4_ (24%)	11.295215
*T*_5_ (25 °C)	1.9088716	P_5_ (25%)	11.555012

**Table 19:** Derivative Business Model.

Classification		Details	Target	Advantages
**Business Model 1**	Mass-Customization-based Intelligent BEMS	Create a derivative revenue model by linking meaningful data between homes and other buildings	Building owners Users	Saving time and cost of IoT system deployment
**Business Model 2**	RL-based Experiential BEMS Management	Save energy through RL-based experiential building management	Building owners Residents	Energy saving rate: 25% ROI: 7.14
**Business Model 3**	Intelligent Smart Energy City Guideline	Expand to new regions based on existing experience data	Businesspersons Urban architects	Cost saving rate of IoT deployment: 15.9% ROI: 7.16
